# Evaluation of Virus-Free Manufacture of Recombinant Proteins Using CRISPR-Mediated Gene Disruption in Baculovirus-Infected Insect Cells

**DOI:** 10.3390/vaccines11020225

**Published:** 2023-01-19

**Authors:** Mark R. Bruder, Marc G. Aucoin

**Affiliations:** Department of Chemical Engineering, University of Waterloo, Waterloo, ON N2L 3G1, Canada

**Keywords:** baculovirus, insect cell, CRISPR, virus-like particle, VLP, BEVS, gp64, HIV Gag

## Abstract

The manufacture and downstream processing of virus-like particles (VLPs) using the baculovirus expression vector system (BEVS) is complicated by the presence of large concentrations of baculovirus particles, which are similar in size and density to VLPs, and consequently are difficult to separate. To reduce the burden of downstream processing, CRISPR-Cas9 technology was used to introduce insertion-deletion (indel) mutations within the *Autographa californica* multiple nucleopolyhedrovirus (*Ac*MNPV) *gp64* open reading frame, which encodes the major envelope protein of *Ac*MNPV. After comfirming the site-specific targeting of *gp64* leading to reduced budded virus (BV) release, the *gag* gene of human immunodeficiency virus type 1 was expressed to produce Gag VLPs. This approach was effective for producing VLPs using the BEVS whilst simultaneously obstructing BV release.

## 1. Introduction

Virus-like particles (VLPs) are an emerging class of biotherapeutic modality for delivery of therapeutic cargo such as chemotherapy, protein, and nucleic acid-based drugs, and as antigens for vaccination [1,2]. VLPs are highly ordered structures that typically self-assemble from a single or multiple viral structural proteins to mimic the three-dimensional structure of the natural virus from which the structural proteins are derived. Additionally, VLPs may be enveloped or nonenveloped, and are replication/infection incompetent, as they lack the genetic material of the natural virus. Finally, the particulate structure of VLPs favours uptake by antigen presenting cells and can stimulate robust B cell and T cell-mediated adaptive and innate immune responses [2,3].

The Baculovirus Expression Vector System (BEVS) has many features that make it an attractive platform for VLP production, including ease of manipulation and large capacity for foreign gene insertion that allows simultaneous expression of multiple proteins from the same recombinant BEV (rBEV) [4]. As such, the BEVS is a preferred platform for production of VLPs, and a multitude of studies have reported successful production of VLPs that mimic many enveloped and nonenveloped viruses [4]. Further, several BEVS-produced VLPs have received regulatory approval for human or veterinary use, or are in various stages of clinical development [5,6]. Nevertheless, significant process shortcomings must be addressed to realize the full potential of the BEVS for VLP production; large amounts of progeny virus, proteins, and cell debris resulting from the lytic infection cycle contaminate the supernatant, requiring extensive purification steps to achieve pharmaceutical-grade purity for clinical applications. In addition, enveloped VLPs and baculovirus are often similar in size, density, and have the same constituent membrane proteins, further complicating downstream processing [5].

To reduce the burden of baculovirus contamination on downstream processing, strategies have been devised wherein a gene encoding a baculovirus structural protein required for viral genome packaging, nucleocapsid assembly, or release of budded viruses (BV) is deleted from its genome. To enable initial production of infectious virus seed stocks, a *trans*-complementing cell line, in which the deleted gene is constitutively expressed, is required. The mutant rBEV is then used to infect parental cells (ie., not expressing the essential gene) for production of the recombinant protein/therapeutic. This approach has been used with the *Ac*MNPV *vp80* and *gp64* genes to produce enhanced green fluorescent protein (EGFP) and HIV-1 Gag VLPs, respectively [7,8]. Both VP80 and GP64 proteins have been shown to be essential to produce infectious budded virus. VP80 is a protein expressed late in the infection involved in the packaging of nucleocapsids and their egress from the nucleus toward the exterior of the cell [7], whereas GP64 is a structural protein that is required for host cell receptor binding and propagation of the budded virus from cell to cell [9]. Although these strategies were successful for reducing the contaminating baculovirus in the supernatant, initial propagation of the rBEV to generate the required viral seed stocks is impaired in both systems, and the overall yield of the recombinant protein from the knockout virus (KOV) may have similarly been affected [7,8].

Here, a recently developed approach for generating rBEV KOVs using CRISPR-Cas9 [10] was used to target the *gp64* gene for disruption. After confirming that targeting the *gp64* open reading frame (ORF) resulted in decreased GP64 abundance in infected cells, expression of the green fluorescent protein (GFP) reporter gene was assessed. Consistent with previous reports, disruption of *gp64* reduced progeny virus release but did not affect expression of GFP. Next, production of HIV-1 Gag VLPs was demonstrated with this approach (targeting *gp64* and *vp80*). The yield of Gag VLPs was similar for all rBEVs in Sf9-Cas9 cells and Sf9 cells, further indicating that CRISPR-mediated disruption of structural genes may be an effective strategy for reducing BV release while maintaining high expression of foreign genes.

## 2. Materials and Methods

### 2.1. Cells and Culture Conditions

Development of the Sf9-Cas9 cells was described previously [10]. Sf9 and Sf9-Cas9 cells were passaged as suspension cultures in Gibco SF900 III serum free medium (Fisher Scientific, Whitby, ON, Canada) in a non-humidified 27 ${}^{{}^{\circ}}C$ incubator and shaken at 130 rpm on an orbital shaker. Puromycin (5 μg/mL; Sigma-Aldrich, Oakville, ON, Canada) was routinely added to the Sf9-Cas9 culture for maintenance of expression of the *cas9* gene.

### 2.2. Plasmid Construction

All plasmids used in this study were constructed using the NEBuilder HiFi DNA Assembly Master Mix (New England Biolabs, Whitby, ON, Canada) according to manufacturer’s directions. Primers used for construction of all plasmids were synthesized by Integrated DNA Technologies (IDT; Coralville, IA, USA) and are given in Table 1. The spacer sequences for the sgRNA are given in Table 2.

The plasmid p6.9GFP-sgRNA, which encodes the p6.9GFP reporter cassette and SfU6-sgRNA for targeting Cas9, has been described previously [10]. Briefly, to construct the p6.9-GFP-encoding CRISPR transfer plasmids, first the coding region of the p10 gene, including upstream and downstream sequences to include its endogenous promoter and 3’ UTR, was amplified from AcMNPV genomic DNA and inserted into pACUW51. The p10 ORF was then replaced with the gfp gene, and the SfU6-sgRNA fragment was inserted downstream to derive p10GFP-sgRNA. Finally, the p6.9 promoter region was amplified from AcMNPV genomic DNA and inserted in place of the p10 promoter sequence in p10GFP-sgRNA to yield p6.9GFP-sgRNA. Inverse PCR was used to exchange the spacer sequence region on plasmid p6.9GFP-sgRNA with those specific to the *gp64* or *vp80* ORF [11]. To generate the transfer plasmids encoding the HIV-1 *gag* gene, the *gfp* ORF was replaced with the *gag* gene from the plasmid pAdCMV5-gagGFP [12] using PCR and NEBuilder HiFi DNA Assembly as described previously [10].

### 2.3. Recombinant Baculovirus Generation, Amplification, and Quantification

Transfer plasmids for rBEV generation were co-transfected with flashBACGOLD™ (Oxford Expression Technologies Ltd., Oxford UK) genomic DNA to Sf9 cells using Escort IV transfection reagent (Sigma-Aldrich) according to manufacturer’s directions. Supernatant from each transfection was harvested 4–5 days post transfection and used to infect suspension Sf9 cultures (∼$1.5\times 10^{6}$ cells/mL) at low multiplicity of infection (MOI) for 3–4 days to amplify the rBEV to higher infectious viral titer (IVT). Following one more round of amplification, the rBEV IVT was quantified using end-point dilution assay (EPDA). Briefly, Sf9 cells were diluted to a density of $2.0\times 10^{5}$ cells/mL and 100 μL was seeded to each well of a 96-well plate (Fisher Scientific). Separately, the rBEV was serially diluted ($10^{-2}$ to $10^{-8}$) in fresh SF900 III medium and 10 μL of each dilution was added, in 12 replicates, to the 96-well plate. Plates were incubated for 6–7 days at 27 ${}^{{}^{\circ}}C$, after which wells were scored according to visualization of green fluorescence using a fluorescence microscope. Results were converted from TCID_50_ as described previously [10] and reported as plaque forming units per mL (pfu/mL).

### 2.4. Infections

Sf9-Cas9 or Sf9 cells were infected with rBEVs at a density of ∼1.5–2 $\times \;10^{6}$ cells/mL at a MOI of 3 pfu/cell. Samples were harvested at the required times (hours post infection; hpi) wherein cells were centrifuged at 300×g for 10 min and resuspended in 2% paraformaldehyde diluted in phosphate buffered saline (PBS) for ∼30 min prior to analysis by flow cytometry. The cell culture supernatant was kept at 4 ${}^{{}^{\circ}}C$ and cell pellets for western blotting were frozen at −80 ${}^{{}^{\circ}}C$.

### 2.5. Western Blot

Infected cells (∼1.5–2 $\times \;10^{6}$ cells/mL) were collected at ∼20–24 hpi by centrifugation at 500×g for 10 min at 4 ${}^{{}^{\circ}}C$. The cells were lysed in RIPA buffer (Fisher Scientific), quantified by Pierce BCA assay (Fisher Scientific), and ∼10 μg of protein was separated by electrophoresis in 10% TGX Stain-Free precast mini SDS-PAGE gels (Bio-Rad, Mississauga, ON, Canada) according to manufacturer’s directions. After transfer to low fluorescence PVDF membranes, Western blot analysis was performed with anti-GP64 (AcV5, Fisher Scientific) primary antibody and goat anti-mouse IgG HRP secondary (Bio-Rad) and imaged on a ChemiDoc MP Imager (Bio-Rad). The Image Lab software (Bio-Rad) was used for further image processing.

### 2.6. Immunofluorescence

Infected cells (∼1 × 10${}^{6}$) were collected at ∼12–15 hpi or ∼48 hpi by centrifugation at 300×g for 10 min at 4 ${}^{{}^{\circ}}C$. The cells were washed twice with cold PBS + 0.5% Bovine Serum Albumin (PBS-BSA) and incubated with anti-gp64 (AcV1, Fisher Scientific) conjugated to APC diluted in PBS-BSA (1:1000) for ∼30 min on ice. Cells were washed 3 times in PBS-BSA and resuspended finally in 200 μL PBS for analysis by flow cytometry.

### 2.7. Flow Cytometry and Analysis

Fluorescent cells were acquired using a BD Accuri™ C6 Plus flow cytometer (BD Biosciences, San Jose, CA, USA) equipped with 488 nm and 640 nm lasers. Samples were run at the low flow setting and 10,000 events were collected and analyzed using FlowJo^®^ V10 flow cytometry analysis software (FlowJo LLC, Ashland, OR, USA).

### 2.8. Quantification of Baculovirus Particles Using Flow Cytometry

Sample preparation for analysis via flow cytometry was described previously [13]. Briefly, samples were diluted in PBS and fixed with paraformaldehyde for 1 h, subjected to one freeze-thaw cycle, and incubated with Triton X-100 to permeabilize the membrane. The nucleic acid stain SYBR Green I was added and incubated at 80 ${}^{{}^{\circ}}C$ for 10 min in the dark to stain double stranded DNA (dsDNA). After cooling on ice, the samples were analyzed via flow cytometry. Flow-Set Fluorospheres (Beckman Coulter, Mississauga, ON, Canada) were used for calibration and all samples were run in triplicate.

### 2.9. Quantification of Gag-VLPs with Enzyme-Linked Immunosorbent Assay (ELISA)

The supernatants of Sf9 and Sf9-Cas9 cells infected with Gag-expressing rBEVs were harvested by centrifugation at 1000×g for 10 min and filter sterilized with a 0.2 μm syringe filter. Gag-VLPs were quantified using the HIV-1 p24 ELISA Kit (Xpress Bio Life Science, Frederick, MD, USA) according to manufacturer’s directions. The absorbance was measured using a Synergy 4 hybrid microplate reader (BioTek, Winooski, VT, USA) at a wavelength of 450 nm. An HIV-1 p24 protein standard of known concentration was used to calculate the Gag concentration and estimate VLP yield.

## 3. Results

### 3.1. Targeting the gp64 ORF Is Site Specific

**Figure 1:**
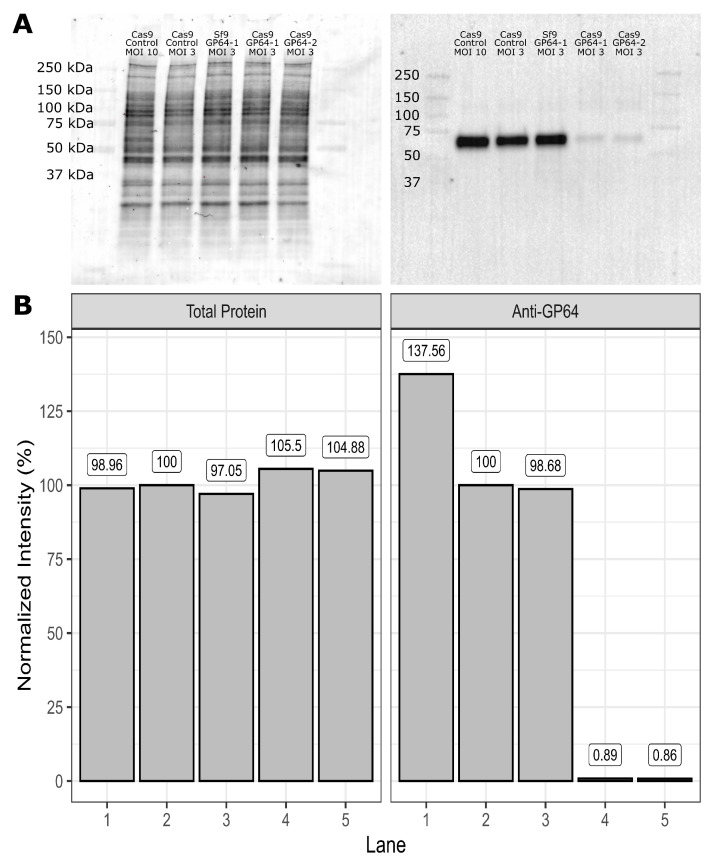
**CRISPR-mediated disruption of *****gp64***** is target specific.** (**A**) Western blot of infected cell lysates with AcV5 anti-GP64 antibody. Left panel: total protein on PVDF membrane after transfer from SDS-PAGE gel. Right panel: PVDF membrane after probing with anti-GP64 AcV5 monoclonal antibody revealed intense bands corresponding to the 4 kDa GP64 protein in lanes 1–3, and very faint bands in lanes 4 and 5. (**B**) Semi-quantitative western blot analysis of GP64 abundance in infected cell lysates. Left panel: Normalized intensity of total protein in each lane of PVDF membrane from (**A**). Lane 2 was selected as the reference for total protein normalization. Right panel: Relative abundance of GP64 determined using total protein normalization. The columns 1–5 in (**B**) correspond to the labeled lanes in (**A**). All samples were taken at 48 hpi.

**Figure 2:**
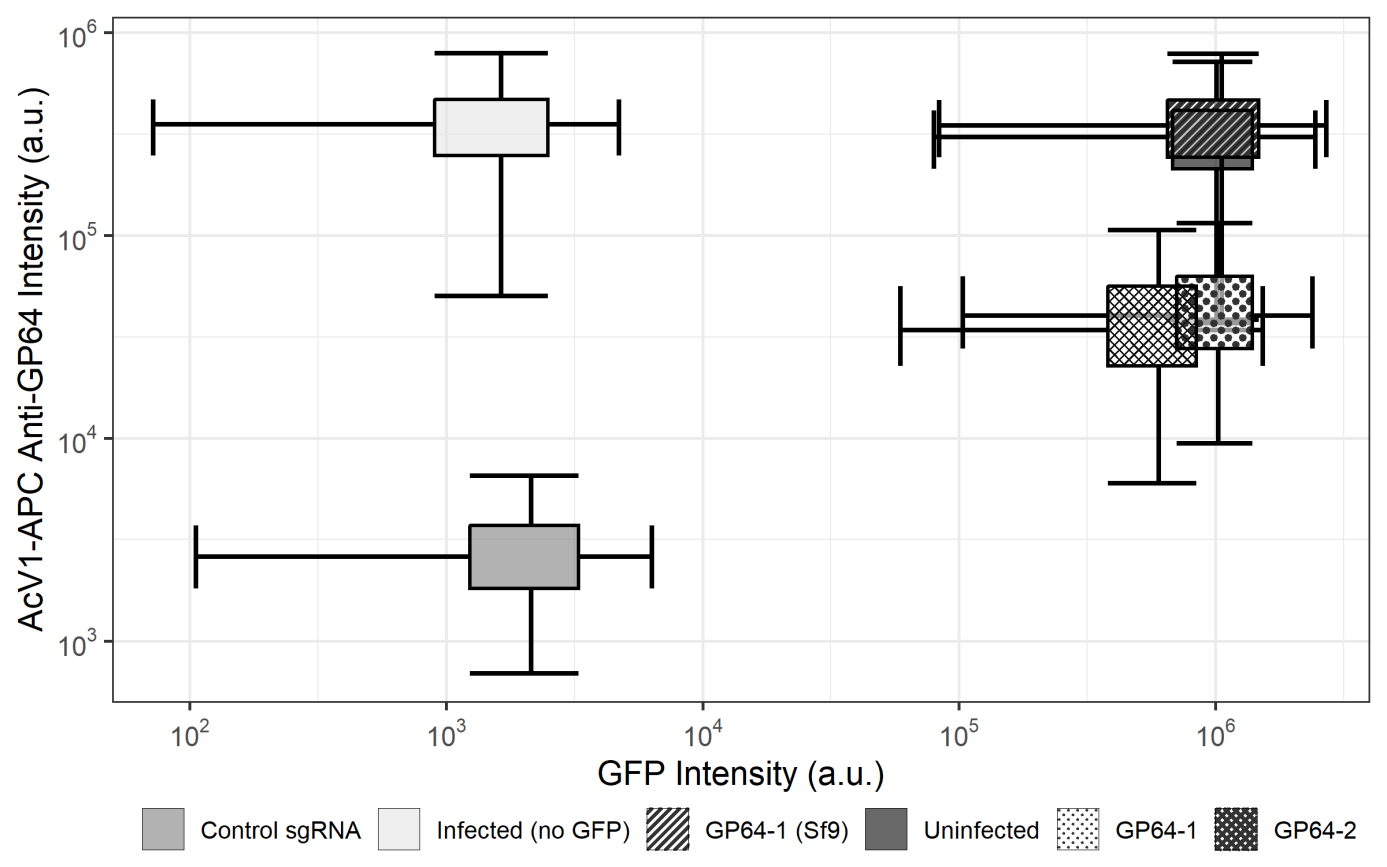
**CRISPR-mediated disruption of *****gp64***** reduces GP64 abundance in the membrane of Sf9-Cas9 cells compared to parental Sf9 cells.** Fluorescence intensity for control and *gp64*-targeted rBEVs expressing the reporter GFP (*x*-axis) and stained with APC-conjugated anti-GP64 AcV1 monoclonal antibody (*y*-axis) represented as a 2-dimensional boxplot. The width and height of the boxes represent the interquartile range (IQR) for the GFP and RFP distributions, respectively. The length of the whiskers are 1.5×IQR. Uninfected control: uninfected cells; Infected Control: Infected with non-fluorescent control rBEV; Control: Sf9-Cas9 cells infected with untargeted sgRNA; GP64-1/GP64-2: Sf9-Cas9 cells infected with *gp64*-targeted sgRNAs; GP64-1 (Sf9): parental Sf9 cells infected with GP64-1 rBEV. All samples were taken at 48 hpi.

Initial experiments were conducted to confirm that sgRNAs designed to target the *gp64* gene were target-specific and resulted in the disruption of progeny virus release. Accordingly, the abundance of GP64 protein was analyzed by western blot and immunofluorescence staining in the cell membrane. Analysis of cell lysates from infected cells revealed that GP64 present in Sf9-Cas9 cells infected with rBEVs targeting the *gp64* ORF was reduced to ∼1% compared to Sf9-Cas9 cells infected with control rBEVs. Parental Sf9 cells infected with the GP64-1 rBEV, on the other hand, showed GP64 levels indistinguishable from the control (Figure 1). Detection of GP64 in the plasma membrane of infected cells similarly revealed reduced fluorescence consistent with lower GP64 abundance in Sf9-Cas9 cells but not parental Sf9 cells (Figure 2). Taken together, these data indicate the sgRNAs designed to target the *gp64* ORF result in decreased abundance of GP64 protein.

### 3.2. Cas9-Mediated Disruption of gp64 Impacts Progeny Virus Production but Not Late Gene Expression

**Figure 3:**
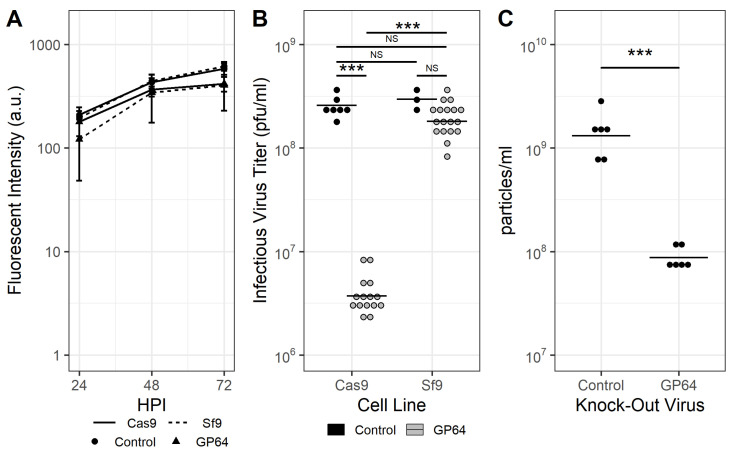
**GP64-disrupted KOVs show reduced IVT and total particle concentration in the supernatant, but unaffected late gene expression.** (**A**) Expression of the *gfp* gene from the viral late p6.9 promoter was similar for all rBEVs in both Sf9-Cas9 and Sf9 cells, however (**B**) IVT, and (**C**) total particle concentration was significantly reduced for control and *gp64*-targeting rBEVs in Sf9-Cas9 but not parental Sf9 cells. Results in panel (**C**) are for Sf9-Cas9 cells only. Solid line: Sf9-Cas9 cells; dashed line: Sf9 cells; untargeted control (circles) and *gp64*-targeted (triangles) rBEVs. All samples were taken at 48 hpi. *** *p* < 0.001.

Sf9-Cas9 cells infected with rBEVs encoding a *gfp* reporter gene transcribed from the late p6.9 gene promoter and sgRNAs targeting the *gp64* gene resulted in significant reduction of infectious viral titer (IVT) at 48 hpi compared to the untargeted control. Specifically, the mean IVT for control rBEVs in Sf9-Cas9 cells was ∼$2.65\times 10^{8}$ pfu/mL whereas the IVT for the Δ*gp64* KOV was $4.03\times 10^{6}$ pfu/mL. Conversely, Sf9 cells infected with the same rBEVs yielded IVTs that were indistinguishable from each other ($3.03\times 10^{8}$ pfu/mL and $1.93\times 10^{8}$ pfu/mL for control and *gp64*-targeting sgRNAs, respectively) and similar to the untargeted control rBEV in Sf9-Cas9 cells (Table 3). Analysis of cell culture supernatants at 8–12 hpi yielded IVT of ∼2.1–6.9 × 10${}^{4}$ pfu/mL for all rBEVs in both cell lines, indicating virus uptake was similar for all rBEVs in Sf9-Cas9 and Sf9 cells (data not shown). Additionally, late gene expression appeared to be unaffected as there were small but insignificant differences in fluorescence intensity between control and *gp64*-disrupted rBEVs in both Sf9 and Sf9-Cas9 cells (Figure 3A,B). Finally, to confirm that this approach resulted in significant reduction of total particles in the supernatant as opposed to only IVT, analysis of cell culture supernatants by flow cytometry revealed that particle concentration was reduced ∼90% compared to the untargeted control rBEV in Sf9-Cas9 cells (Figure 3C). This evidence suggests that CRISPR-mediated disruption of the *gp64* gene resulted in a reduction of particles in culture supernatants but does not significantly impact late gene expression.

### 3.3. Production of HIV-1 Gag VLPs

**Figure 4:**
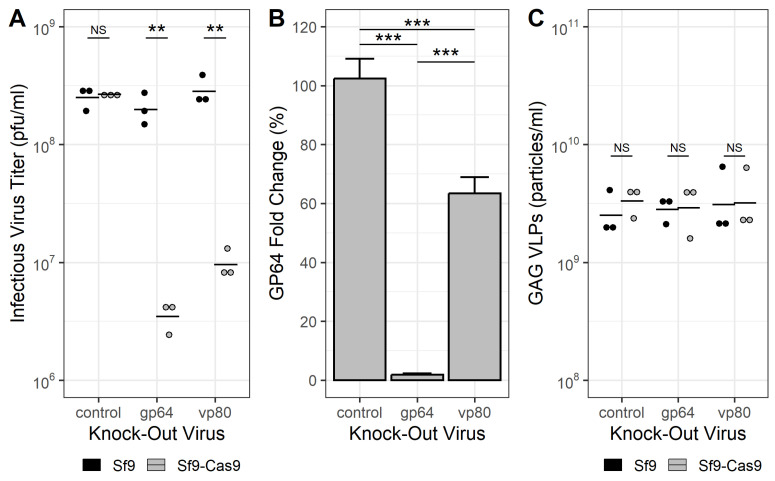
**Disruption of the *****gp64***** gene reduces rBEV contamination more than disruption of the *****vp80***** gene.** (**A**) *gp64*-KOVs produce less budded virus than *vp80*-KOVs as measured by IVT in Sf9-Cas9 cells but were both similar to the control with parental Sf9 cells. (**B**) Abundance of GP64 in infected cell membranes of Sf9-Cas9 cells was significantly lower for *gp64* KOVs compared to parental Sf9 cells. Targeting the *vp80* gene also had an apparent effect on GP64 abundance in the membrane. (**C**) Yield of HIV-1 Gag VLPs was not significantly different for any KOVs in either Sf9-Cas9 or Sf9 cells. All samples were taken at 48 hpi. ** *p* < 0.01, *** *p* < 0.001.

In light of these results, the *gfp* reporter gene was replaced with the HIV-1 *gag* gene to investigate the production of Gag VLPs with this system. In addition to targeting the *gp64* ORF for disruption, rBEVs expressing *gag* and sgRNAs targeting the *vp80* ORF were also prepared. Infecting Sf9-Cas9 cells with rBEVs resulted in ∼99% reduction of IVT for rBEVs targeting the *gp64* and ∼94% for the *vp80* target (Figure 4A) compared to the same infections in Sf9 cells. Similarly, GP64 in the plasma membrane was reduced by ∼99% for the *gp64*-targeting sgRNAs. Interestingly, targeting the *vp80* ORF resulted in ∼35% reduction in GP64 (via immunofluorescence analysis) compared to control infections in Sf9 cells, indicating that targeting the *vp80* ORF may have an impact on GP64 expression (Figure 4B). Finally, quantification of Gag VLPs by ELISA indicated VLP yields of ∼3–6 $\times \;10^{9}$ particles/mL for all rBEVs in both Sf9 and Sf9-Cas9 cells. These yields were not significantly different from each other, indicating that production of Gag VLPs was not impaired by disruption of either *gp64* or *vp80* genes (Figure 4C).

## 4. Discussion

Although the production of virus-like particles in insect cells using BEVs is well-established, the presence of high concentrations of baculovirus particles that are co-produced along with VLPs in the culture supernatant, complicates and increases the cost of the downstream processing [5]. This is especially true for enveloped VLPs that bud out of the cell via the cytoplasmic membrane.

To address this drawback, strategies have been devised to reduce or eliminate progeny baculovirus production through the targeted deletion of genes encoding structural proteins that are required for BV release, called knockout viruses (KOVs) [5,7,8]. This strategy requires the development of a *trans*-complementing cell line to enable replication of the rBEV. However, this approach may be less effective for rBEV seed production, and foreign gene expression and overall yield is reportedly lower than with conventional, wildtype rBEV systems [7,8]. We recently developed a novel system for producing KOVs based on CRISPR-Cas9 mediated introduction of indel mutations in the *Ac*MNPV genome [10]. This system is able to disrupt progeny BV release and/or reduce late gene expression through targeted disruption of several *Ac*MNPV genes. Targeting *gp64* or the *vp80* gene, which encodes the nucleocapsid-associated protein VP80, with this approach resulted in reduced BV release but did not appear to significantly impact expression of the *gfp* reporter gene.

To assess this strategy for its utility as an effective production platform for VLP production with concomitant reduced BV release, we again targeted the *Ac*MNPV *gp64* gene for disruption. To this end, the abundance of GP64 in infected cell lysates and in the membrane of infected cells was measured. Our results indicated ∼99% and ∼90–95% reduction of GP64 in lysates and in the membrane of infected Sf9-Cas9 cells, respectively. Importantly, the abundance of GP64 in Sf9 cells infected with rBEVs targeting *gp64* was indistinguishable from control infections, indicating that disruption of GP64 expression was the result of CRISPR-mediated targeting of the *gp64* ORF.

Next, the effect of targeting *gp64* on late gene expression and progeny BV release was measured. Disruption of GP64 resulted in >98% and ∼94% reduction of IVT and total particles/mL, respectively. This data is consistent with a previous report in which BV release was reduced by ∼50–98% for different *gp64* gene truncations [14]. Similarly, GP64 appeared to be undetectable for the Δ*gp64* KOV via western blot, however direct quantification of BV in the supernatant was not conducted in that report [8]. For late gene expression, our results indicated that expression of the *gfp* reporter gene was not significantly affected by *gp64* disruption. Although the median fluorescence intensity was slightly lower for *gp64*-targeting rBEVs compared to the control, this difference in expression was similar for both Sf9-Cas9 and Sf9 cell lines. This data could indicate that variability between individual virus stocks may have accounted for these differences as opposed to decreased late gene expression as a result of CRISPR-mediated targeting. Nevertheless, these differences were not statistically significant. This is an important result, as previous reports indicated that high MOIs were required for similar EGFP yields between Δ*vp80* KOV and the control virus [7], whereas high MOIs were not necessary with the system developed here. Furthermore, in previous studies, production of Gag VLPs appeared to be lower via western blot analysis between the Δ*gp64* KOV and the control [8]; and with the system developed here, the difference was negligible.

Finally, we assessed the production of HIV-1 Gag VLPs with concomitant reduced BV contamination. The HIV-1 *gag* ORF encodes a 55 kDa polyprotein (Pr55 or Gag) that is processed into several proteins, including the 17 kDa matrix protein (p17 or MA), the 24 kDa capsid protein (p24 or CA), and the 7 kDa nucleocapsid protein (p7 or NC) [15]. Expression of Gag alone is sufficient for assembly and budding of VLPs, and several studies have demonstrated production of pseudotyped and non-pseudotyped Gag VLPs in the BEVS and in uninfected insect cells [8,16,17,18,19,20,21]. In addition to targeting *gp64*, rBEVs with sgRNAs targeting the *vp80* ORF were prepared in order to compare VLP production using both of these strategies. Similar to previous results, targeting the *gp64* ORF resulted in significant reduction of GP64 abundance in the plasma membrane of infected cells and IVT. The IVT of *vp80*-disrupted rBEVs was also significantly reduced compared to control infections in Sf9 cells. Unexpectedly, immunofluorescence staining of GP64 in the plasma membrane of infected cells was observed to be lower in Sf9-Cas9 cells compared to Sf9 cells, suggesting that disruption of VP80 expression may impact GP64 production. Reduced GP64 was not observed by western blot analysis of cell lysates infected with a Δ*vp80* KOV previously [7], however staining of GP64 in the membrane of those cells was not conducted. On the other hand, analysis of VP39 by western blot indicated lower abundance in cells infected with the Δ*gp64* KOV [8]. The results here do not appear to be associated with off-site targeting of the Cas9/sgRNA ribonucleoprotein complex, as 2 other sgRNAs targeting the *vp80* ORF showed similar results (data not shown). Similarly, there were insignificant differences between GP64 measurements in the cell membranes infected with control or *vp80*/*gp64*-targeted rBEVs (data not shown). As such, this observation appears to be the result of a potential and as yet unreported interaction between *vp80* disruption and GP64 expression, and may require further scrutiny to assess this relationship. Nevertheless, both of these strategies were successful for producing Gag VLPs with concomitant reduction in rBEV contamination. Importantly, although the estimated yield of VLPs by p24 ELISA was lower compared to a control (ie., untargeted rBEV expressing the *gag* gene), yields of VLPs were similar in Sf9-Cas9 and Sf9 cells for all of the rBEVs, suggesting that these results might be due to variance among virus seed stocks as opposed to the strategy itself.

## 5. Conclusions

In this report, CRISPR-mediated disruption of the *gp64* gene was assessed. After confirming that this strategy resulted in target specific obstruction of GP64 and reduced BV release, production of HIV-1 Gag VLPs was assessed and compared with a similar strategy in which the *vp80* ORF was targeted for disruption. Both strategies resulted in high level production of VLPs along with reduced rBEV contamination in culture supernatants. This strategy may be impactful for simplifying the purification of recombinant proteins and other complex biologics such as VLPs, and may be an improvement over previously reported strategies in which initial virus seed production was impaired and overall yield may be impacted.

## Figures and Tables

**Table 1:** Primers used in this study.

Plasmid Construct	Sequence (5′-3′)	Use (Template)
Retarget sgRNAs	gttttagagctagaaatagcaagttaaaataagg	retarget sgRNA † (fwd primer)
	cggtggtcgagcacga	retarget sgRNA † (rev primer)
p6.9GAG-sgRNA	cgaccccagcagccagtaaggcgcgccatgaatc	p6.9-sgRNA backbone
	catgtttaaattgtgtaatttatgtagctgtaatttttacc	
	acagctacataaattacacaatttaaacatgggcgccagagcc	HIV-1 gag ORF
	cgattcatggcgcgccttactggctgctggggtcg	

†: spacer sequence appended to 5′ end of sequence.

**Table 2:** Protospacer sequences for CRISPR targets.

Gene	Protospacer Sequence (5′-3′)	PAM	Strand
GP64-1	GGAAACGCTGCAAAAGGACG	TGG	Template
GP64-2	GTTGTAGTCCGTCTCCACGA	TGG	Nontemplate
VP80-1	GCCCGCCGCAATCGCCGCCG	CGG	Template
VP80-2	TCGCTGGATGTTACCCGCGG	CGG	Nontemplate

**Table 3:** Summary of fluorescence intensity and virus quantification data for rBEVs in Sf9 and Sf9-Cas9 cells at 48 hpi. A minimum n = 3 was used in all cases.

rBEV	Sf9-Cas9			Sf9		
	FL. Intensity (au)	IVT (pfu/mL)	Particles/mL	FL. Intensity (au)	IVT (pfu/mL)	Particles/mL
Control	434±3.96	$2.65\pm 0.59\times 10^{8}$	$1.47\pm 0.76\times 10^{9}$	443±6.90	$3.03\pm 0.74\times 10^{8}$	-
GP64	367±5.70	$4.03\pm 1.89\times 10^{6}$	$8.93\pm 2.16\times 10^{7}$	342±13.70	$1.93\pm 0.65\times 10^{8}$	-

## Data Availability

The datasets generated during and/or analysed during the current study are available from the corresponding author on reasonable request.

## Abbreviations

The following abbreviations are used in this manuscript:

AcMNPV	*Autographa californica* multiple nucleopolyhedrovirus
APC	allophycocyanin
au	arbitrary units
BV	budded virus
BEVS	baculovirus expression vectors system
rBEV	recombinant baculovirus expression vector
eGFP	enhanced Green fluorescent protein
EPDA	end-point dilution assay
GFP	green fluorescent protein
hpi	hours post infection
hpt	hours post transfection
IQR	interquartile range
IVT	infectious virus titer
KOV	knockout virus
MOI	multiplicity of infection
ORF	open reading frame
PVDF	polyvinylidene difluoride
VLP	virus-like particle
